# Freezing of gait in Parkinson’s disease with glucocerebrosidase mutations: prevalence, clinical correlates and effect on quality of life

**DOI:** 10.3389/fnins.2023.1288631

**Published:** 2023-11-28

**Authors:** Ruwei Ou, Chunyu Li, Qianqian Wei, Kuncheng Liu, Junyu Lin, Tianmi Yang, Yi Xiao, Qirui Jiang, Yangfan Cheng, Yanbing Hou, Lingyu Zhang, Wei Song, Xueping Chen, Xiaohui Lai, Huifang Shang

**Affiliations:** ^1^Department of Neurology, Laboratory of Neurodegenerative Disorders, National Clinical Research Center for Geriatrics, West China Hospital, Sichuan University, Chengdu, Sichuan, China; ^2^Department of Neurology, West China Hospital, Sichuan University, Chengdu, Sichuan, China

**Keywords:** Parkinson’s disease, freezing of gait, GBA1 mutation, quality of life, gene mutation

## Abstract

**Objectives:**

Mutations in glucocerebrosidase (*GBA1*) can change the clinical phenotype of Parkinson’s disease (PD). This study aimed to explore the clinical characteristics of freezing of gait (FOG) in PD patients with *GBA1* mutations.

**Methods:**

A whole-exome sequencing analysis was used to identify the *GBA1* mutations (pathogenic or likely pathogenic) and exclude other PD-related gene mutations. A forward binary logistic regression model was conducted to identify the associated factors of FOG. The stepwise multiple linear regression analysis models were used to explore the effect of FOG on quality of life.

**Results:**

The prevalence of FOG in patients with *GBA1* mutations (30/95, 31.6%) was significantly higher than those in patients without *GBA1* mutations (152/760, 20%) (*p* = 0.009). A higher (i.e., worse) Unified PD Rating Scale part III score (OR = 1.126, 95%CI = 1.061–1.194, *p* < 0.001) and a lower (i.e., worse) Montreal Cognitive Assessment score (OR = 0.830, 95%CI = 0.713–0.967, *p* = 0.017) were significantly associated with FOG in PD patients with *GBA1* mutations. The presence of FOG was significantly associated with the decreased (i.e., worse) score of PD Questionnaire 39 after adjustment for sex, age, disease duration, motor score, and non-motor score (B = 14.981, *p* = 0.001).

**Conclusion:**

FOG is a relatively common disabling symptom in PD patients with *GBA1* mutations, which is affected by motor disability and cognitive decline. Quality of life is reduced in patients with FOG and *GBA1* mutations.

## 1 Introduction

Freezing of gait (FOG) is a common symptom in Parkinson’s disease (PD) (Rogers, 1996), affecting approximately 60% of patients (Lamberti et al., 1997). FOG is regarded as a brief, episodic absence or marked reduction of forwarding progression of the feet despite the intention to walk (Giladi and Nieuwboer, 2008), which mainly occurs as start hesitation, upon turning or approaching a destination, or sometimes even when walking in an open walkway (Stern et al., 1980). FOG significantly impairs mobility and increases the risk of falls among PD patients (Giladi, 2001), which greatly reduces the quality of life (QoL) of patients (Ou et al., 2014).

Glucocerebrosidase (*GBA1*) mutations are common in PD and represent one of the most important risk factors yet discovered for PD (Schapira, 2015). Mutations in *GBA1* can modify the clinical phenotype of PD (Davis A. A. et al., 2016). Compared to non-carriers, individuals with *GBA1* mutations have been reported to exhibit earlier onset symptoms, are more likely to have affected relatives, and increased chances to have atypical clinical manifestations (Sidransky et al., 2009). Furthermore, *GBA1* mutation carriers with PD are on a trajectory to cognitive decline (Liu et al., 2016), and they are more prone to present with the postural instability gait difficulty phenotype compared with non-carriers (Malek et al., 2018). However, the clinical symptoms of FOG in PD patients with *GBA1* mutations remain largely unknown. Detailed descriptions regarding the clinical characteristics based on specific genotypes are important for precision medicine. The aim of this study is therefore to explore the clinical characteristics of FOG in a group of PD patients with *GBA1* mutations.

## 2 Methods

### 2.1 Study patients

This study was approved by the Ethics Committee of West China Hospital of Sichuan University. All patients signed informed consent. A total of 95 PD patients with *GBA1* mutations from the Department of Neurology, West China Hospital of Sichuan University were recruited between October 2010 and September 2021. The diagnosis of PD was based on the Unified Kingdom PD Society Brain Bank Clinical Diagnostic Criteria for PD (Hughes et al., 1992). Patients without *GBA1* mutations, patients with severe dementia or hearing loss, and those who refused to be interviewed were excluded from this study. Additionally, we included 760 PD patients without *GBA1* mutations as controls. These patients came from the same research center at the same time and underwent whole-exome sequencing (WES). All recruited patients conducted brain MRI scans to exclude other neurological disorders, such as cerebrovascular disease, brain tumors, and encephalitis.

### 2.2 Clinical assessments

Clinical data including age, age of onset, gender, disease duration, years of education, body mass index (BMI), treatment regimen, and motor complications were collected through in-person interviews by neurologists specializing in PD. The Unified PD Rating Scale (UPDRS) part III (Goetz et al., 2007) and Hoehn and Yahr (H&Y) stage (Hoehn and Yahr, 1967) were used to evaluate the motor severity. The quality of life (QoL) of PD patients was assessed using PD Questionnaire 39 (PDQ-39), which consists of eight domains (Mobility, Activities of daily life, Emotional well-being, Stigma, Social support, Cognitions, Communication, and Bodily discomfort) (Jenkinson et al., 1995). Non-motor symptoms (NMS) were measured using the Non-Motor Symptoms Scale (NMSS) (Wang et al., 2009). Cognition was evaluated utilizing the Frontal Assessment Battery (FAB) (Dubois et al., 2000) and Montreal Cognitive Assessment (MoCA) (Nasreddine et al., 2005). Depression and anxiety were assessed using the Hamilton Depression Rating Scale (HAMD) (24 items) [20] and the Hamilton Anxiety Rating Scale (HAMA) [21], respectively.

Freezing episodes during the visit were observed by experienced neurologists or reported by the patients themselves, along with their family members or caregivers when FOG occurred at home or anywhere outside of the hospital. Patients were identified as experiencing FOG (freezers) based on a score (≥1) from item 1.3 of the FOG Questionnaire, which asked: “Do you feel that your feet get glued to the floor while walking, making a turn, or when trying to initiate walking?” [22]. The responses provided by patients were verified by their relatives or caregivers and cross-checked with clinical records for data accuracy.

### 2.3 Molecular genetic analysis of *GBA1* mutations

Information regarding *GBA1* mutations in PD patients is presented in Supplementary Table S1. Patients carrying *GBA1* mutations (pathogenic or likely pathogenic) were identified and excluded if they carried mutations in other PD-related genes through WES analysis. Genomic DNA was extracted from peripheral blood leukocytes using standard phenol-chloroform procedures. A total of 5ug DNA was fragmented into an average size of 350 bp with a Covaris LE220-plus focused ultrasonicator, and the DNA library was constructed with the KAPA Library Amplification Kit. Then WES was performed routinely on the Illumina NovaSeq 6,000 system following manufacturer’s instructions. Pathogenicity categorization followed the American College of Medical Genetics and Genomics (ACMG) guidelines (Richards et al., 2015).

### 2.4 Statistical analyses

Data analyses were performed using the Statistical Package for the Social Sciences (SPSS) version 22.0 for Windows. All statistical tests were two-tailed, with *p* values < 0.05 considered statistically significant. Frequencies and descriptive statistics were utilized to summarize the clinical features of samples. Data was reported as percentages for categorical variables and mean ± standard deviation (SD) for continuous variables. The Student’s *T* test, Chi-square test, Fisher exact test, and Wilcoxon rank-sum test were employed to compare clinical data between PD patients with and without FOG.

A binary logistic regression model was utilized to explore potential factors associated with FOG. The presence or absence of FOG was used as the dependent variable. The following parameters: sex (male/female), age, disease duration, motor fluctuation (yes/no), dyskinesia (yes/no), education, as well as the UPDRS III, HAMD, HAMA, MoCA, FAB, and NMSS scores, were set as covariables.

The stepwise multiple linear regression analysis models were used to explore the effect of FOG on QoL. The models were initially performed with adjustments for sex, age, disease duration, UPDRS III score, and NMSS score (Model 1), followed by additional adjustments for motor fluctuation and dyskinesia (Model 2), and further adjustments for HAMD, HAMA, MoCA, and FAB scores (Model 3). A collinearity test was conducted which revealed no collinearity among these variables.

## 3 Results

In the current study, we included 95 PD patients with *GBA1* mutations and 30 of them (31.6%) reported FOG. Individuals with *GBA1* mutations showed younger age than those without *GBA1* mutations (48.9 ± 10.5 years vs. 51.6 ± 12.1 years, *p* = 0.020). No significant differences in male proportion (50.5% vs. 50.7%, *p* = 0.981), age of onset (44.8 ± 10.3 years vs. 46.7 ± 12.1 years, *p* = 0.096), and disease duration (4.0 ± 3.8 years vs. 4.8 ± 5.1 years, *p* = 0.066) were observed between the two groups. The prevalence of FOG in patients with *GBA1* mutations was significantly higher than those in patients without *GBA1* mutations (31.6% vs. 20%, *p* = 0.009).

**Figure 1:**
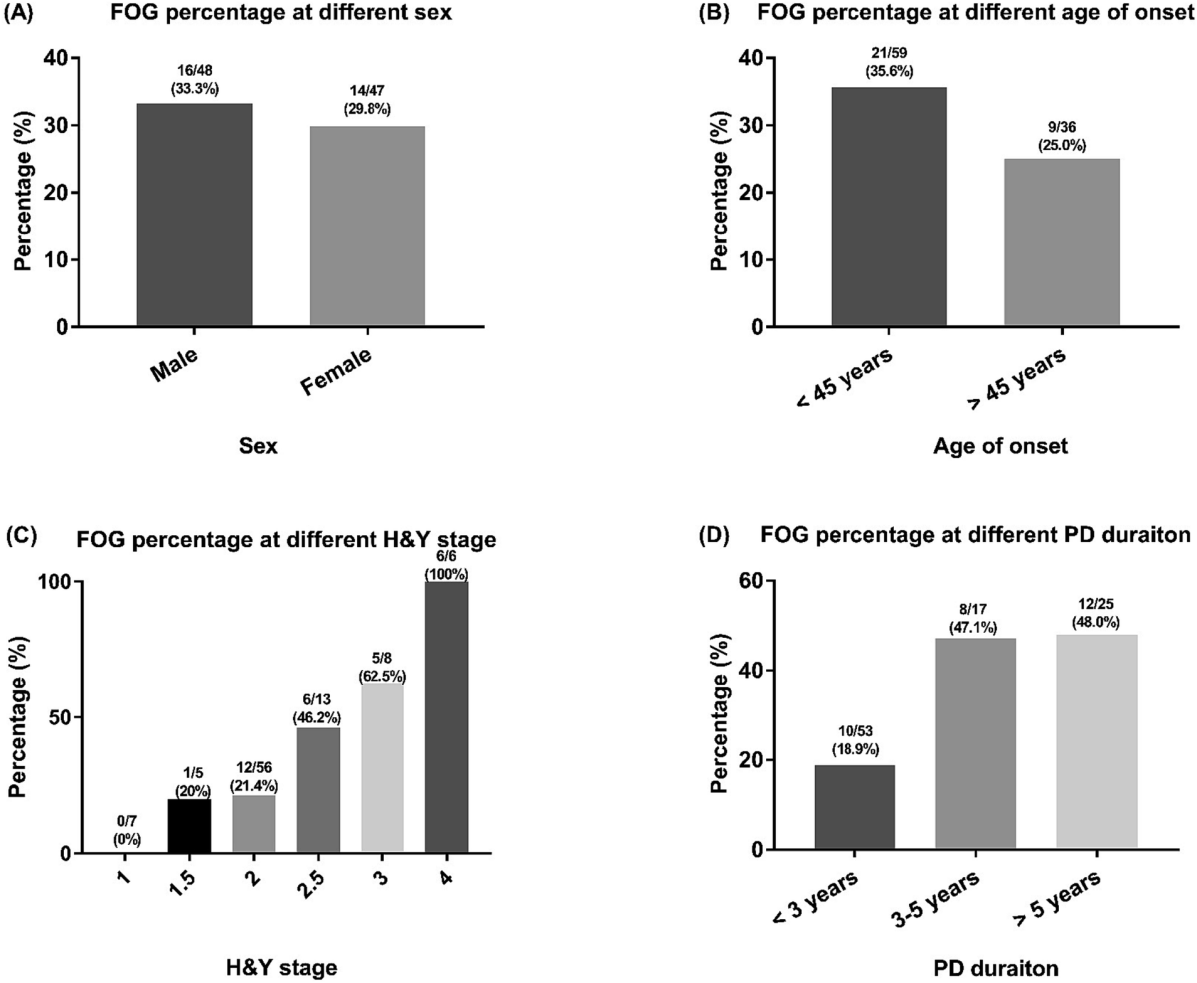
The FOG percentages of included PD patients with GBA1 mutations. FOG, freezing of gait; PD, Parkinson’s disease; GBA1, glucocerebrosidase. Comparisons of the percentage of FOG among PD patients across different categories, including sex **(A)** age at onset **(B)** H&Y stage **(C)** and PD duration **(D)** groups.

The prevalence of FOG in male patients was 33.3% and the prevalence of FOG in female *GBA1* patients was 29.8% (Figure 1A). In *GBA1* patients with an age of onset <45 years, 35.6% patients reported FOG; while in patients with an age of onset >45 years, 25.0% patients were experiencing FOG (Figure 1B). Patients with higher H&Y stage or longer disease duration reported a higher prevalence of FOG (Figures 1C,D).

The demographic and clinical features of the PD patients with *GBA1* mutaitons are listed in Table 1. The freezers showed a significantly higher (i.e., worse) UPDRS III score (*p* < 0.001), greater (i.e., worse) H&Y stage (*p* < 0.001), more frequent motor fluctuation (*p* = 0.015), higher (i.e., worse) NMSS score (*p* = 0.001), higher (i.e., worse) HAMD score (*p* < 0.001), higher (i.e., worse) HAMA score (*p* = 0.035), lower (i.e., worse) MoCA score (*p* < 0.001), and lower (i.e., worse) FAB scores (*p* = 0.044) than the non-freezers. The mean age, age of onset, disease duration, LEDD, and sex distribution were not significantly different between patients with and without FOG in *GBA1* carriers (All *p* > 0.05).

**Table 1:** Demographic and clinical features of PD patients with *GBA1* mutations.

	Total (*n* = 95)	Freezers (*n* = 30)	Non-freezers (*n* = 65)	*p*-value
Education years (years)	10.1 ± 4.2	9.0 ± 4.1	10.6 ± 4.1	0.097
BMI (kg/m^2^)	22.4 ± 2.9	22.5 ± 2.8	22.4 ± 3.1	0.926
Male sex (%)	48 (50.5%)	18 (60.0%)	30 (46.2%)	0.210
Age (years)	49.0 ± 10.4	50.8 ± 10.7	48.1 ± 10.2	0.234
Age of onset (years)	44.9 ± 10.2	45.9 ± 10.3	44.5 ± 10.2	0.526
Disease duration (years)	4.0 ± 3.8	4.9 ± 4.1	3.6 ± 3.7	0.127
LEDD (mg/day)	343.8 ± 329.3	416.1 ± 360.0	310.4 ± 311.3	0.147
Use of levodopa (%)	58 (61.1%)	21 (70.0%)	37 (56.9%)	0.224
Use of dopamine agonist (%)	34 (35.8%)	9 (30.0%)	25 (38.5%)	0.424
Use of MAO-B inhibitor (%)	4 (4.2%)	0	4 (6.2%)	0.304
Use of amantadine (%)	28 (29.5%)	9 (30.0%)	19 (29.2%)	0.939
Use of benzhexol (%)	4 (4.2%)	1 (3.3%)	3 (4.6%)	1.000
Use of COMT inhibitor (%)	5 (5.3%)	3 (10.0%)	2 (3.1%)	0.322
UPDRS III	29.8 ± 14.0	41.5 ± 15.1	24.4 ± 9.4	<0.001^*^
H&Y stage	2.0 (0.5)	2.5 (1.0)	2.0 (0)	<0.001^*^
Fluctuation (%)	18 (18.9%)	10 (33.3%)	8 (12.3%)	0.015^*^
Dyskinesia (%)	11 (11.6%)	5 (16.7%)	6 (9.2%)	0.292
NMSS	34.1 ± 38.2	58.3 ± 53.9	22.9 ± 20.6	0.001^*^
HAMD	11.4 ± 9.3	17.4 ± 10.7	8.6 ± 7.0	<0.001^*^
HAMA	7.9 ± 7.7	10.9 ± 10.4	6.5 ± 5.6	0.035^*^
MoCA	24.9 ± 4.1	22.4 ± 4.6	26.0 ± 3.3	<0.001^*^
FAB	16.2 ± 2.3	15.4 ± 2.9	16.6 ± 1.9	0.044^*^

PD, Parkinson’s disease; GBA1, glucocerebrosidase; BMI, body mass index; LEDD, levodopa equivalent daily dose; MAO-B, monoamine oxidase type B; COMT, catechol-O-methyltransferase; UPDRS, Unified Parkinson’s Disease Rating Scale; H&Y stage, Hoehn and Yahr stage; NMSS, Non-Motor Symptoms Scale; HAMD, Hamilton Depression Rating Scale; HAMA, Hamilton Anxiety Rating Scale; MoCA, Montreal Cognitive Assessment; FAB, Frontal Assessment Battery. ^*^Significant difference.

The PDQ-39 results for the patients with and without FOG in PD patients with *GBA1* mutations are listed in Table 2. The group experiencing freezing episodes demonstrated significantly higher (i.e., worse) total scores on the PDQ-39 (*p* < 0.001) as well as higher (i.e., worse) scores in the domains of mobility (*p* < 0.001), activities of daily life (*p* < 0.001), emotional well-being (*p* = 0.006), stigma (*p* = 0.029), communication (*p* < 0.001), and bodily discomfort (*p* = 0.020) compared to those without freezing episodes.

**Table 2:** The PDQ-39 scores in PD patients with *GBA1* mutations.

PDQ-39	Freezers	Non-freezers	*p*-value
Total score	58.7 ± 25.5	28.3 ± 20.2	<0.001^*^
Mobility	18.3 ± 10.6	5.4 ± 5.9	<0.001^*^
Activities of daily life	11.5 ± 6.9	4.4 ± 4.6	<0.001^*^
Emotional well-being	8.8 ± 5.6	5.5 ± 5.2	0.006^*^
Stigma	6.4 ± 4.8	4.3 ± 4.2	0.029^*^
Social support	2.0 ± 2.5	1.1 ± 2.2	0.071
Cognitions	4.5 ± 3.8	3.3 ± 2.5	0.108
Communication	3.3 ± 2.6	1.2 ± 1.9	<0.001^*^
Bodily discomfort	3.9 ± 3.1	2.4 ± 2.1	0.020^*^

PD, Parkinson’s disease; PDQ-39, Parkinson’s Disease Questionnaire 39; GBA1, glucocerebrosidase. ^*^Significant difference.

The potential factors associated with FOG in PD patients with *GBA1* mutations are outlined in Table 3. The binary logistic regression model revealed that a higher UPDRS III score (OR = 1.126, 95%CI = 1.061–1.194, *p* < 0.001) and a lower MoCA score (OR = 0.830, 95%CI = 0.713–0.967, *p* = 0.017) were both significantly associated with FOG in PD patients with *GBA1* mutations.

**Table 3:** Associated factors of FOG in PD patients with *GBA1* mutations.

	OR	95%CI	*p*-value
MoCA	0.830	0.713–0.967	0.017^*^
UPDRS III	1.126	1.061–1.194	<0.001^*^

The model adjusted for sex, age, disease duration, motor fluctuation, dyskinesia, and education, as well as HAMD, HAMA, NMSS, and FAB scores. FOG, freezing of gait; PD, Parkinson’s disease; GBA1, glucocerebrosidase; MoCA, Montreal Cognitive Assessment; UPDRS, Unified PD Rating Scale. ^*^Significant difference.

The impact of FOG on the QoL among patients with *GBA1* mutations is presented in Table 4. Multiple regression analysis models indicated that the presence of FOG remained significantly associated with decreased QoL after adjusting for sex, age, disease duration, motor score, and non-motor score in Model 1, 2, and 3 (*p* < 0.05).

**Table 4:** Association between FOG and PDQ-39 score in PD with *GBA1* mutations.

	PDQ-39 score			
FOG	Unstandardized coefficients (B)	Standardized coefficient (β)	*t*	*P*-value
Model 1	18.930	0.339	4.428	<0.001^*^
Model 2	11.141	0.199	2.385	0.019^*^
Model 3	14.981	0.268	3.594	0.001^*^

Model 1 adjusted for sex, age, disease duration, UPDRS III score, and NMSS score. Model 2 adjusted for motor fluctuation and dyskinesia in addition to Model 1. Model 3 adjusted for HAMD, HAMA, MoCA, and FAB scores in addition to Model 2. PD, Parkinson’s disease; FOG, freezing of gait; PDQ-39, PD Questionnaire 39; GBA1, glucocerebrosidase; UPDRS, Unified PD Rating Scale; NMSS, Non-Motor Symptoms Scale; HAMD, Hamilton Depression Rating Scale; HAMA, Hamilton Anxiety Rating Scale; MoCA, Montreal Cognitive Assessment; FAB, Frontal Assessment Battery. ^*^Significant difference.

## 4 Discussion

To the best of our knowledge, this is the first study to investigate the clinical characteristics of FOG in PD patients with *GBA1* mutations. In the current study, FOG was observed in nearly 30% of PD patients with *GBA1* mutations, which is a higher prevalence compared to those without *GBA1* mutations. We found that FOG in individuals carrying *GBA1* mutation was influenced by motor severity and cognitive decline. In addition, we also found that FOG significantly decreased the QoL for PD patients with *GBA1* mutations.

In the current study, high frequency of the “severe” p.L444P/p.L483P *GBA1* mutations were included, which accounted for almost half (45/95, 47.4%) of the pathogenic/likely pathogenic *GBA1* cases. In a previous multi-ethnic Asian cohort with PD, p.L444P/p.L483P (11/496, 2.2%) was also detected as the most common variant in patients with *GBA1* mutations (Lim et al., 2022). This suggests that our data are representative of the Asian PD population. Studies from Western populations usually find other *GBA1* variants to be more common, such as p.E326K/p.E365K, p.T369M/p.T408M, and p.N370S/p.N409S (Malek et al., 2018), which on the other hand are usually absent in Asian populations (Lim et al., 2022). Therefore, our findings in the current study require further confirmation in the European PD population.

In our previous study conducted on 474 general PD patients with an average duration of 4.8 ± 4.0 years, we reported a high prevalence of FOG among the Chinese PD population at 46.6% (Ou et al., 2014). Although our current study included patients with shorter disease duration (mean 4.0 ± 3.8 years), approximately one-third of those with *GBA1* mutations experienced FOG. The prevalence of FOG observed in our study aligns closely with the result from a recent meta-analysis reporting a weighted prevalence for overall early-stage PD (≤ 5 years) at 37.9% (Zhang et al., 2021). A previous small sample size study involving only 22 *GBA1* mutation carriers revealed that FOG was present in as many as 72.2% of PD patients who had an average disease duration of 12.1 years (Da Silva et al., 2017). Discrepancies between these studies may be attributed to differences in sample sizes, inclusion criteria, and genetic backgrounds. Furthermore, our findings indicate that the incidence of FOG was significantly higher in the *GBA1* population than in the non-*GBA1* population. This novel finding requires further confirmation through large-sample and multi-center studies.

In the present study, after adjusting for age, disease duration, H&Y stage, and NMSS, our PD patients with FOG exhibited a reduced QoL, particularly in terms of bodily discomfort. These findings are consistent with those reported in an Israeli study (Moore et al., 2007), highlighting the significant impact of FOG on QoL of PD patients beyond its effect on gait. Currently, only a limited number of studies have investigated how the mechanism of FOG affects QoL, and a previous study suggested that the episodic nature of FOG may play a role (Moore et al., 2007).

Our *GBA1* mutation carriers with FOG demonstrated more severe motor disability and more frequent motor complications in PD, aligning with our previous observational study (Ou et al., 2014) as well as several studies conducted on Caucasian populations (Giladi et al., 1992; Lamberti et al., 1997; Giladi et al., 2001). The association between motor severity and FOG in our *GBA1* mutation population is consistent with the findings from general PD populations in America and Germany [8, 24]. In addition, a prospective study revealed that *GBA1* mutations were associated with accelerated motor decline in PD (Davis M. Y. et al., 2016). However, contrary to previous studies conducted on Caucasian populations [5, 6, 8, 23, 24], we did not find an association between disease duration and FOG. Additionally, no correlation was identified between dopaminergic drugs and FOG among patients with *GBA1* mutations—a finding inconsistent with some prior studies involving the general PD population [6, 8, 24].

In addition, the association between FOG and cognitive decline in our sample is consistent with several previous studies (Amboni et al., 2008, 2010; Nantel et al., 2012), which have demonstrated that FOG was linked to executive dysfunction and visuospatial deficits. The pathogenesis of FOG remains unclear. A resting-state fMRI study (Tessitore et al., 2012b) revealed that disruption in connectivity within the “executive attention” and visual neural networks may be associated with FOG in the general PD population. One voxel-based morphometry study found an association between FOG and posterior gray matter atrophy (Tessitore et al., 2012a). Another voxel-based morphometry found that PD patients with FOG exhibited frontal and parietal atrophy, suggesting the involvement of executive dysfunction and perception deficits (Kostic et al., 2012). Our studies highlight the significance of cognitive circuits and motor pathways in relation to FOG among patients with *GBA1* mutations. Further investigations into its pathophysiology can provide valuable insights.

Some limitations should be acknowledged. (1) There may exist recall bias. (2) The cross-sectional analysis conducted in this study can only indicate possible associations but cannot reveal causality. (3) We did not determine whether patients experienced FOG during ON or OFF states. (4) The sample size was relatively small. (5) Sanger sequencing was not performed to verify *GBA1* mutations. (6) The function and severity of certain *GBA1* mutations still require further confirmation.

## 5 Conclusion

FOG is a relatively common disabling symptom observed among PD patients with *GBA1* mutations. Motor disability and cognitive decline are likely contributors to the occurrence of FOG among carriers of *GBA1* mutations. QoL is diminished for individuals with *GBA1* mutations. Our findings provide similar results regarding FOG in patients with *GBA1* mutations compared to previous reports on the overall PD population.

## Data availability statement

The original contributions presented in the study are included in the article/Supplementary material, further inquiries can be directed to the corresponding authors.

## Ethics statement

The studies involving humans were approved by Ethics Committee of West China Hospital of Sichuan University. The studies were conducted in accordance with the local legislation and institutional requirements. Written informed consent for participation in this study was provided by the participants’ legal guardians/next of kin.

## Author contributions

RO: Conceptualization, Data curation, Formal analysis, Funding acquisition, Investigation, Methodology, Project administration, Resources, Software, Supervision, Validation, Visualization, Writing – original draft, Writing – review & editing. CL: Data curation, Investigation, Methodology, Validation, Writing – review & editing. QW: Data curation, Investigation, Validation, Writing – review & editing. KL: Data curation, Investigation, Methodology, Validation, Writing – review & editing. JL: Data curation, Investigation, Writing – review & editing. TY: Investigation, Methodology, Writing – review & editing. YX: Investigation, Methodology, Writing – review & editing. QJ: Investigation, Methodology, Writing – review & editing. YC: Investigation, Methodology, Writing – review & editing. YH: Investigation, Methodology, Writing – review & editing. LZ: Investigation, Methodology, Writing – review & editing. WS: Investigation, Methodology, Writing – review & editing. XC: Investigation, Methodology, Writing – review & editing. XL: Validation, Visualization, Writing – review & editing, Conceptualization, Data curation, Methodology, Project administration, Software, Supervision. HS: Conceptualization, Funding acquisition, Resources, Supervision, Validation, Writing – review & editing.
