# Supplementary material for: Freezing of gait in Parkinson’s disease with glucocerebrosidase mutations: prevalence, clinical correlates and effect on quality of life
Source: Front Neurosci. 2023 Nov 28;17:1288631. doi: 10.3389/fnins.2023.1288631 (PMC10713814; doi:10.3389/fnins.2023.1288631)
Supplement: Supplementary file 1 [file Table_1.DOCX]

**Supplementary Table S1** Information of *GBA* mutations in PD patients

| Number of patients | Zygosity | Exon | cDNA change | Amino acid change | ClinVar database | Severity |
| --- | --- | --- | --- | --- | --- | --- |
| 45 | Het. | 11 | c.1448T>C | p.Leu483Pro | Pathogenic | Severe |
| 5 | Het. | 10 | c.1342G>C | p.Asp448His | Pathogenic | Severe |
| 5 | Het. | 6 | c.476G>A | p.Arg159Gln | Likely pathogenic | Severe |
| 4 | Het. | 7 | c.754T>A | p.Phe252Ile | Pathogenic | Severe |
| 3 | Het. | 7 | c.721G>A | p.Gly241Arg | Pathogenic | Severe |
| 2 | Het. | 7 | c.703T>C | p.Ser235Pro | Pathogenic | Unknown |
| 2 | Het. | 8 | c.928A>G | p.Ser310Gly | Likely pathogenic | Mild |
| 2 | Het. | 10 | c.1297G>A | p.Val433Met | Likely pathogenic | Unknown |
| 2 | Het. | 8 | c.943G > T | p.Val315Phe | Likely pathogenic* | Unknown |
| 2 | Het. | 10 | c.1279_1280insCCCT  GAACCCCG | p.P426_  E427insALNP | Likely pathogenic* | Unknown |
| 1 | Het. | 9 | c.1085C>T | p.Thr362Ile | Pathogenic | Unknown |
| 1 | Het. | 7 | c.680A>G | p.Asn227Ser | Pathogenic | Severe |
| 1 | Het. | Intron3 | c.115+1G>A | - | Likely Pathogenic | Unknown |
| 1 | Het. | Intron4 | c.307+1G>A | - | Likely Pathogenic* | Unknown |
| 1 | Het. | Intron4 | c.308-1G>A | - | Likely Pathogenic* | Unknown |
| 1 | Het. | Intron10 | c.1388+3G>C | - | Likely Pathogenic* | Unknown |
| 1 | Het. | 6 | c.475C>T | p.Arg159Trp | Pathogenic | Severe |
| 1 | Het. | 10 | c.1246G>A | p.Gly416Ser | Pathogenic | Severe |
| 1 | Het. | 10 | c.1292A>G | p.Asn431Ser | Likely pathogenic* | Unknown |
| 1 | Het. | 7 | c.681T>G | p.Asn227Lys | Likely pathogenic | Severe |
| 1 | Het. | 7 | c.680A>G | p.Asn227Ser | Pathogenic | Severe |
| 1 | Het. | 6 | c.544C>T | p.Gln182* | Likely pathogenic* | Severe |
| 1 | Het. | 8 | c.928A>G | p.Ser310Gly | Likely pathogenic | Mild |
| 1 | Het. | 9 | c.1018G>A | p.Ala340Thr | Likely pathogenic | Unknown |
| 1 | Het. | 10 | c.988T > A | p.Trp330Arg | Likely pathogenic* | Unknown |
| 1 | Het. | 9 | c.1048C>T | p.His350Tyr | Likely pathogenic | Unknown |
| 1 | Het. | 9 | c.1174delC | p.Arg392Trp | Pathogenic | Unknown |
| 1 | Het. | 6 | c.506T>G | p.Ile169Ser | Likely pathogenic* | Unknown |
| 1 | Het. | 7 | c.675G>C | p.Lys225Asn | Likely pathogenic* | Unknown |
| 1 | Het. | 9 | c.1143_1145del | p.381_382del | Likely pathogenic* | Unknown |
| 1 | Het. | 7 | c.712G>A | p.Gly238Arg | Likely pathogenic* | Unknown |
| 1 | Het. | 10 | c.1255G>A | p.Asp419Asn | Likely pathogenic* | Mild |
| 1 | Het. | 4 | c.226T>G | p.Phe76Val | Likely pathogenic* | Unknown |

* This rare variant was classified as likely pathogenic by our previous study (published in 2020) ^[^[^1^](#_ENREF_1)^]^.

Abbreviations: Het, heterozygous; PD, Parkinson’s disease.

**Reference:**

1. Chen Y, Gu X, Ou R*, et al.* Evaluating the Role of SNCA, LRRK2, and GBA in Chinese Patients With Early-Onset Parkinson's Disease. *Mov Disord* 2020;35:2046-2055. doi: 10.1002/mds.28191.
